# Radiation dose to the eye of physicians during radio frequency catheter ablation: a small-scale study

**DOI:** 10.1007/s11845-024-03802-6

**Published:** 2024-10-05

**Authors:** Yoshiaki Morishima, Koichi Chida, Hiroo Chiba, Koji Kumagai

**Affiliations:** 1 Department of Radiological Technology, Tohoku Medical and Pharmaceutical University Hospital, 1-12-1 Fukumuro, Miyagino-Ku, Sendai, 983-8512 Japan; 2 Department of Radiological Technology, Tohoku University School of Health Sciences, Sendai, 980-8575 Japan; 3 Cardiac Center, Tohoku Medical and Pharmaceutical University Hospital, Sendai, 983-8512 Japan

**Keywords:** DOSIRIS, Glass badge, Lens equivalent dose, Radiation protection, RFCA

## Abstract

**Background:**

Radio frequency catheter ablation (RFCA), a treatment for arrhythmia, requires a long fluoroscopy time that increases the radiation exposure dose to the physician, particularly to the lens of the eye. It is recommended that a lens-specific dosimeter such as DOSIRIS® is used to measure the dose to the lens.

**Aims:**

In this study, we investigated whether conventional glass badges can be used as an alternative to lens dosimeters.

**Methods:**

The doses to the lenses of two physicians (physician A, main operator; physician B, assistant; physician B was further away from the patient than physician A) were measured for 126 RFCA procedures performed over a 6-month period (fluoroscopy rate of 3.0 p/s with use of a ceiling-hanging shield).

**Results:**

The cumulative value measured by a lens dosimeter attached to the inside of Pb glasses (0.07-mm dose equivalent) next to the left eye was 4.7 mSv for physician A, and 0.8 mSv for physician B. The reading on the glass badge worn on the left side of the neck was 4.7 mSv for physician A and 1.3 mSv for physician B. Lens dosimeter and glass badge values showed a good correlation for the left eye and left neck (*r* = 0.86, *p* < 0.01).

**Conclusions:**

We show that glass badges may be a viable alternative to lens-equivalent dosimetry when using low-pulse fluoroscopy and a ceiling-hanging shield.

## Introduction

Arrhythmia is one of the most common heart diseases, and underlying medical conditions can pose a risk of triggering atrial fibrillation [1, 2]. Interventional radiology procedures, such as radio frequency catheter ablation (RFCA), are commonly used to treat arrhythmias [3, 4]. Recently, techniques such as three-dimensional cardiac mapping have become widespread, and it has become possible to accurately identify the cauterized part [5, 6].

Interventional radiology procedures involve long fluoroscopic exposure times, with significant radiation doses to physicians and patients [7–12]. However, the introduction of technologies such as RFCA mapping has been reported to reduce fluoroscopy time [13–21].

A statement from the International Commission on Radiological Protection (ICRP) at the 2011 Seoul conference suggested that eye tissue reactions such as cataracts can occur at radiation doses lower than previous dose limits. In its 118th publication in 2012, the ICRP proposed a new dose limit for the lens of the eye [22]. This dose limit was 20 mSv per year averaged over a 5-year period, with no more than 50 mSv in any 1 year. The ICRP also published a “Statement on Tissue Response” that proposed that the absorbed dose threshold for preventing cataract induction from ionizing radiation should be no more than 0.5 Gy for acute or chronic exposure [22]. Before this, the dose thresholds for preventing cataracts were 5 Gy for acute exposure and 8 Gy or higher for chronic exposure [23].

The International Atomic Energy Agency issued guidelines (TECDOC-1731) in 2014 following the ICRP recommendations. These guidelines recommend that the dose to the lens of the eye should be measured close to the eye.

Many studies have investigated the lens-equivalent dose (LED) to physicians during interventional radiology procedures [24–26]. In Japan, regulation for the LED started in April 2021. The ICRP 103 guidelines (2007) recommend that the LED should be monitored using a 3-mm dose equivalent [Hp (3)] value [27]. However, most facilities currently monitor the LED with a 0.07-mm dose equivalent [Hp (0.07)], and not a Hp (3) value [28].

Measurement of the LED by the DOSIRIS® eye dosimeter (Institute for Radiation Protection and Nuclear Safety, Clamart, France) has been reported in several studies [29–33], but there are very few reports on the use of the DOSIRIS in RFCA procedures. In one previous study, the dose to the eye lens of the physician tended to be overestimated by neck glass badge measurements, and the evidence suggested that eye lens doses may not be estimated accurately by neck glass badges [7]. It is also possible that not all physicians use a ceiling-hanging shield correctly.

In this study, we used the DOSIRIS and a glass badge to measure the LED during RFCA procedures performed over a 6-month period (3-mm dose equivalent). Using the DOSIRIS as a reference dosimeter, we investigated whether physicians could use a conventional glass badge as an alternative to lens dosimeters during RFCA.

## Materials and methods

### Facilities

A digital cine single-plane angiography unit (Infinix Celeve-i8000; Canon Medical Systems, Tokyo, Japan) with an under-tube X-ray tube system was used for the procedures monitored in this study. Pulsed fluoroscopy (3.0 pulses/s) was performed with a 17-cm flat panel detector with a grid of 13:1 and 70 lines/cm. The control system for this equipment automatically sets the kilovolts and milliamperes of the X-ray exposure.

**Fig. 1:**
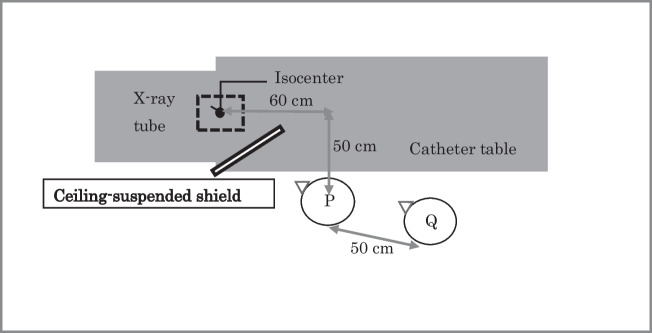
Position of physicians relative to X-ray tube and ceiling-suspended shield during radio frequency catheter ablation in the cardiac catheterization laboratory. P, physician A (main operator) position; Q, physician B (assistant) position

**Fig. 2:**
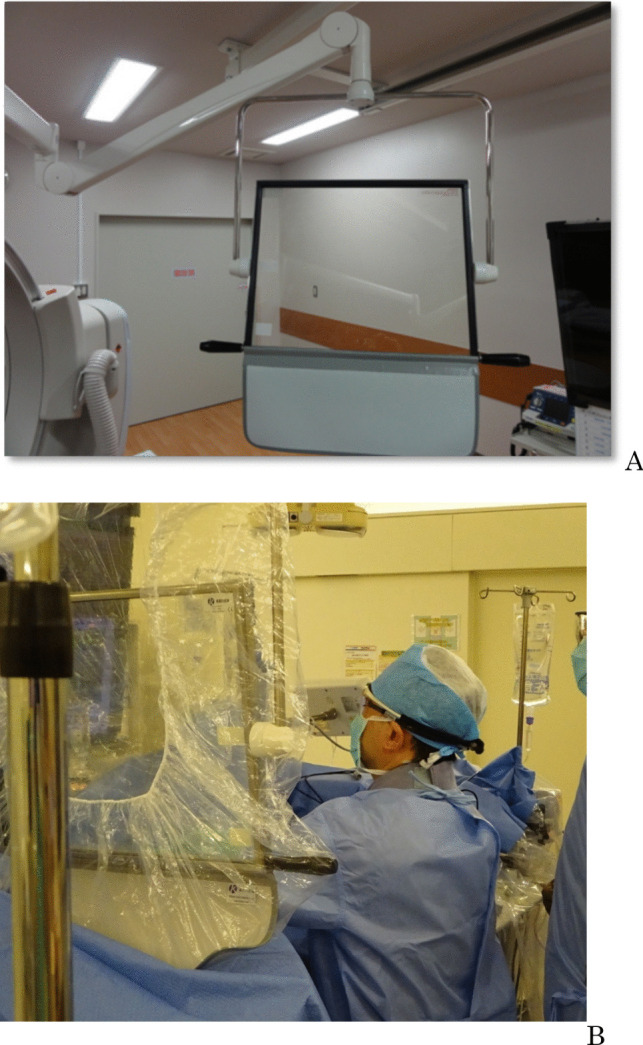
**A** Ceiling-suspended shield (2.0-mm lead equivalent for acrylic plate, 0.5-mm lead equivalent for lead sheet). **B** Use of lead shielding plate during radio frequency catheter ablation

The positions of the physicians relative to the X-ray tube and ceiling-suspended shield (2.0-mm lead equivalent for acrylic plate, 0.5-mm lead equivalent for lead sheet) used during the RFCA procedure are shown in Figs. 1 and 2. Points P and Q indicate the positions of physician A (main operator) and physician B (assistant), respectively.

### Dosimeter

The DOSIRIS consists of a thermo-luminescent dosimeter sensor (^7^LiF:Mg, Ti), a 3-mm polypropylene capsule with a measurement range of 0.1 mSv to 1 Sv, and an adjustable headset.

**Fig. 3:**
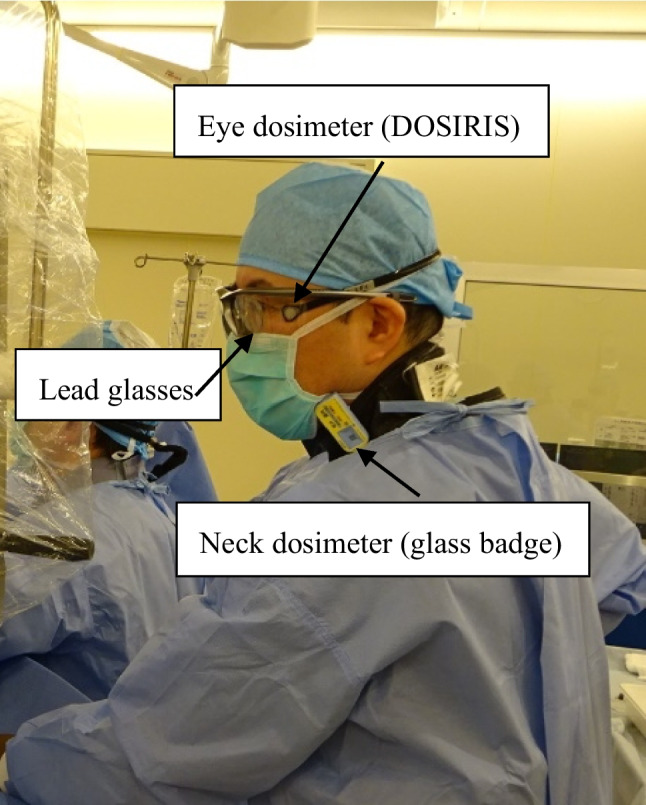
Positions of dosimeters and lead glasses. Physicians wore a DOSIRIS lateral to the left eye inside of the lead glasses (0.07-mm lead). Personal dosimeters (glass badges) were worn at the left side of the neck outside the protective lead apron

Both participants wore the DOSIRIS at a lateral angle to the left eye (Fig. 3) and used lead glasses (0.07-mm lead; Toray Medical Co., Ltd., Tokyo, Japan). The doses estimated by the DOSIRIS were compared with those recorded by a personal dosimeter (glass badge: measurement range of 0.1 mSv to 2 Sv) worn outside the protective apron on the left side of the neck. The personal dosimeter was a silver-activated phosphate glass dosimeter (0.07-mm dose equivalent, Hp (0.07), Glass Badge; Chiyoda Technol Corporation, Tokyo, Japan). The personal dosimeters were used only for this study.

### Participants

Two physicians working at Tohoku Medical and Pharmaceutical University Hospital participated in this study. They wore protective aprons (0.35-mm lead equivalent) during the RFCA procedures, and the measurements were performed over 6 months (from November 2020 to April 2021).

### Statistical analysis

A Pearson correlation test was used to determine whether the doses estimated by the neck glass badge were linearly related to those estimated by the eye dosimeters. Statistical significance was defined as *p* < 0.01. All analyses were performed using SPSS Version 24 (IBM Corp., Armonk, NY, USA).

## Results

### Patient characteristics

During the 6-month study period, 126 RFCA examinations were performed. The 126 RFCA procedures included 100 for atrial fibrillation, 8 for premature ventricular contraction, 7 for paroxysmal supraventricular tachycardia, 6 for atrial flutter, 3 for atrial tachycardia, and 3 for ventricular tachycardia. One patient underwent RFCA for both atrial tachycardia and ventricular tachycardia.

The patients comprised 88 (69.8%) men and 38 (30.2%) women. Their mean age was 63.4 ± 13.7 years, and their mean body weight was 69.3 ± 13.6 kg. The mean fluoroscopy time was 64.0 ± 25.7 min. The mean dose was 524.0 ± 394.9 mGy•cm^2^ (Table 1).

**Table 1:** Patient data (*N*, Male/female, age, body weight, fluoroscopy time, and DAP). There is one duplicate data point for AT and VT

*N*	126
AF (atrial fibrillation)	100
PVC (premature ventricular contraction)	8
PSVT (paroxysmal supraventricular tachycardia)	7
AFL (atrial flutter)	6
AT (atrial tachycardia)	3
VT (ventricular tachycardia)	3
Male/female	88/38 (69.8%/30.2%)
Age (y)	63.4 ± 13.7
Body weight (kg)	69.3 ± 13.6
Fluoroscopy time (min)	64.0 ± 25.7
Dose area product, DAP (mGy・cm^2^)	524.0 ± 394.9

**Fig. 4:**
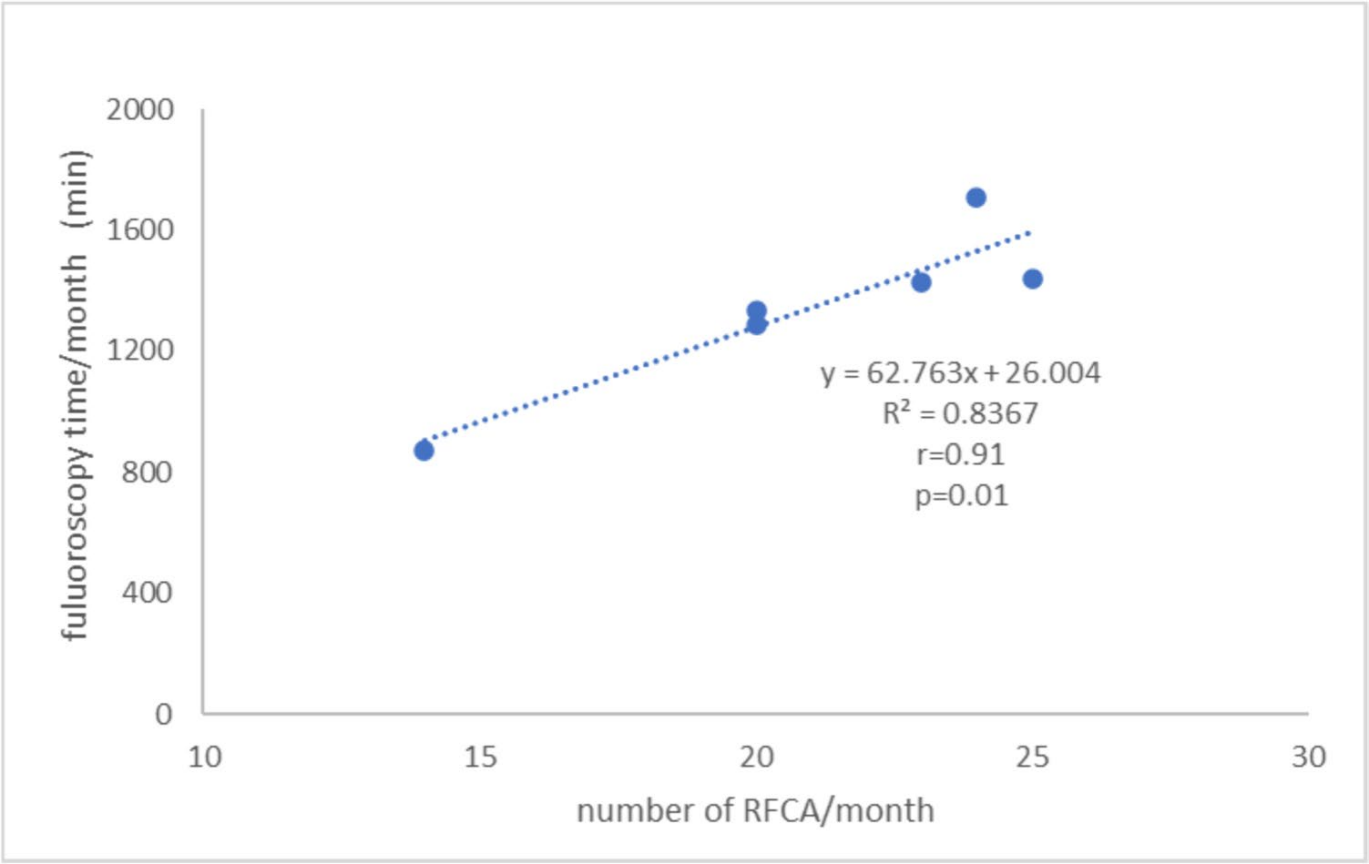
Relationship between total fluoroscopy time per month and number of radio frequency catheter ablation procedures over 6 months. The correlation coefficient was *r* = 0.91 (*p* = 0.01)

Figure 4 shows the relationship between the total fluoroscopy time per month and the number of RFCA procedures. A strong, statistically significant correlation was observed between the fluoroscopy time and the number of RFCA procedures (*r* = 0.91, *p* = 0.01).

### LED to the physicians

Table 2 shows the cumulated LED to the two physicians over 6 months. The LED to physician A at the outer edge of the eye was estimated as 4.7 mSv by the DOSIRIS [Hp (3)] and 4.7 mSv by the glass badge (Hp [0.07]). The estimated doses to physician B at the outer edge of the eye were 0.8 mSv by the DOSIRIS and 1.3 mSv by the glass badge.

**Table 2:** Cumulative LEDs of physicians during RFCA over 6 months. Physician A, main operator, position P; Physician B, assistant, position Q (see Fig. 1)

	Radiation dose (mSv)			
	DOSIRIS Hp (3)		Glass badge Hp (0.07)	
Physician A	4.7		4.7	
Min–max (per month)	0.5–1.0		0.3–1.3	
Physician B	0.8		1.3	
Min–max (per month)	0.1–0.2		0.1–0.3	

**Fig. 5:**
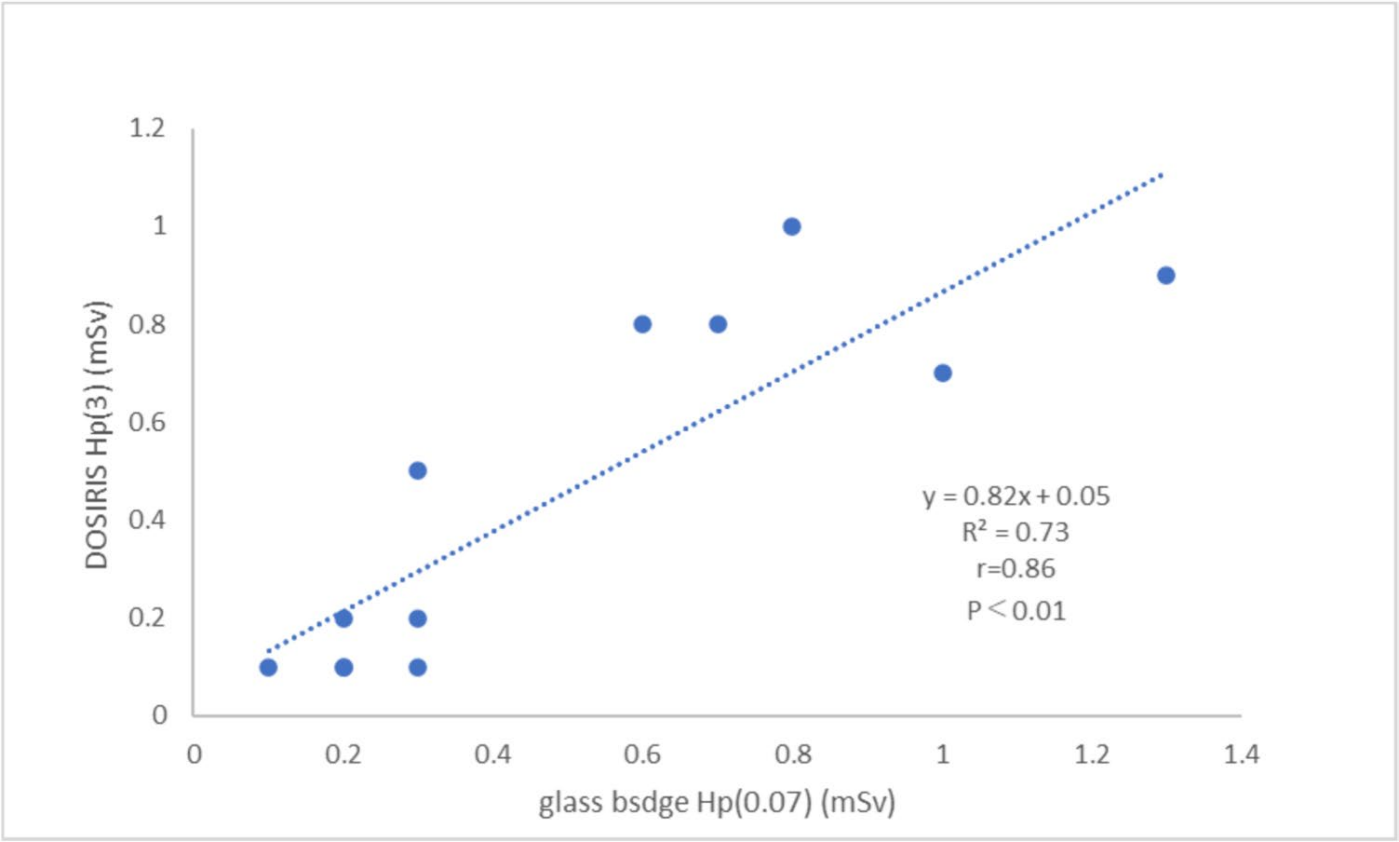
Relationship between DOSIRIS Hp (3) (left eye) and neck glass badge Hp (0.07) (left neck) radiation doses for physicians during radio frequency catheter ablation per month over 6 months. The correlation coefficient between Hp (3) and Hp (0.07) was *r* = 0.86 (*p* < 0.01). The total number of data points is 12, but there is one duplicate data point

Figure 5 shows the relationship between the LEDs using the DOSIRIS [Hp (3)] and the glass badge [Hp (0.07)] for physicians A and B. A strong, statistically significant correlation was observed between the Hp (3) and Hp (0.07) measurements (*r* = 0.86, *p* < 0.01). The total number of data points was 12, but there was one duplicate data point.

## Discussion

We used the DOSIRIS as a dedicated dosimeter to investigate whether a glass badge could be a substitute for LED measurement. There was a very good correlation between the total fluoroscopy time per month and the number of RFCA procedures (*r* = 0.91, *p* = 0.01) (Fig. 4).

Our results showed a good correlation between the LED of physicians measured according to the Hp (3) dose and Hp (0.07) dose (Fig. 5). A study that compared Hp (3) and Hp (0.07) using a slab phantom showed that Hp (0.07) was not underestimated [34].

To protect the crystalline lens during catheter intervention, lead glasses are required in addition to ceiling-suspended shield [35]. The use of lead glasses (0.07-mm lead) in clinical settings can reportedly reduce the radiation dose by approximately 60% [24, 36]. If lead glasses are not used properly, the eye will be exposed to a high dose. Annual exposures in excess of 20 mSv have also been reported [37].

Medical staff such as nurses can stand farther from the patient to protect themselves from scattered radiation. However, physicians must remain close to the patient because they are directly involved in performing the clinical procedures, and the use of an additional lead shielding device is therefore necessary [38–50].

Kato et al. [7] reported that the occupational eye lens doses measured by the DOSIRIS during RFCA procedures were 5.65 to 13.99 mSv over 6 months, which are higher values than those measured in this study. There is a possibility that some physicians may receive more than 20 mSv per year. Therefore, additional radiation protection measures such as lead glasses are necessary.

We consider that the lens dose tended to be lower in the present study because the lead equivalent of the ceiling-suspended shield was higher (2.0-mm lead), and we used low-pulse fluoroscopy (3 pulses/s). Most of the scattered radiation is reportedly generated by patients in angiography units using under-tube X-ray tube systems [49].

The use of a low pulse rate reduces the generation of scattered radiation [43], and ceiling-suspended shield with a high lead equivalent are able to shield most of the scattered radiation. Therefore, we presume that the DOSIRIS and glass badge showed almost the same values even when wearing lead glasses.

Our measurements using the DOSIRIS and glass badges were almost the same, showing a good correlation. We conclude that by effectively using a low pulse rate and ceiling-suspended shield, a glass badge could be an alternative to LED measurement.

## Limitations

We analyzed only a small data sample from a single hospital. Further studies with a larger sample population will be needed to accurately measure the exposure dose to the eyes.

## Conclusions

We considered whether glass badges can replace lens dosimeters during RFCA, using the DOSIRIS as a reference dosimeter. We conclude that although it is recommended to use a dosimeter specifically designed for the lens, a glass badge may be able to replace the lens dosimeter.

## Data Availability

The data that support the findings of this study are available from the corresponding author upon reasonable request.
